# Sex Differences in Jump Capacity and Elastic Index in Table Tennis Players

**DOI:** 10.3390/jfmk10020099

**Published:** 2025-03-21

**Authors:** Jon Mikel Picabea-Arburu, Eñaut Ozaeta-Beaskoetxea

**Affiliations:** 1 Society, Sports and Physical Exercise Research Group (GIKAFIT), Physical Education and Sport Department, Faculty of Education and Sport, University of the Basque Country (UPV/EHU), 01007 Vitoria-Gasteiz, Spain; 2 Bioaraba Health Research Institute, Physical Activity, Exercise, and Health Group, 01004 Vitoria-Gasteiz, Spain; 3 Department of Physical Education and Sport, Faculty of Education and Sport, University of the Basque Country (UPV/EHU), 01006 Vitoria-Gasteiz, Spain; enaut.ozaeta@ehu.eus

**Keywords:** racket sport, explosive strength, lower limbs, vertical jump, horizontal jump

## Abstract

**Background/Aims:** Table tennis performance is influenced by various factors such as technique, tactics, and fitness. Additionally, many shots are executed at high speeds, developing significant levels of explosive strength in the lower extremities. This study aimed to assess the jump capacity and the elasticity index of the lower limbs among young table tennis players based on sex. Additionally, this study assessed leg asymmetries between the dominant and non-dominant limbs during jump tests. **Methods:** A total of 40 players (20 boys and 20 girls), aged 16–18 years, participated in the study. Vertical countermovement jump, squat jump, and horizontal jump tests were conducted to evaluate both vertical and horizontal jumping capacities, as well as leg asymmetries between the dominant and non-dominant limbs. **Results:** Differences were observed in both vertical and horizontal jumps, with male players achieving better results in all jumping capacities. However, female players obtained better values in elastic index. Additionally, significant differences were found between dominant and non-dominant legs in both male and female players. **Conclusions:** Considering that explosive strength is one of the most essential physical capacities in this sport, this information could prove valuable for talent identification, the design of training programs, and the optimization of physical performance monitoring systems in table tennis.

## 1. Introduction

Table tennis is a globally recognized Olympic sport, practiced by millions of competitive and non-competitive players around the world [1,2,3]. This sport has undergone numerous rule changes in recent years, including the introduction of the plastic ball, an increase in the ball’s diameter and weight, a new 11-point scoring system, speed glue prohibition, and restrictions on service techniques, along with others [4]. The aforementioned modifications have significantly changed fundamental aspects of table tennis, including physiological and metabolic demands, game structure, and physical requirements, which differ considerably from those observed in matches played years ago [2,4]. As a result, the game may evolve differently depending on whether it is played by one gender or another. Despite table tennis’ immense popularity, long history, and major regulatory and gameplay changes, there remains a notable lack of scientific studies investigating this sport, particularly in relation to both genders [2].

Table tennis is a racket sport characterized by intermittent physical efforts, with high-intensity and high-speed actions, making it one of the fastest sports in terms of game speed [1,2]. Approximately 4% of the efforts produced during a match depend on anaerobic energy pathways, associated with short duration and high intensity actions. In contrast, the remaining 96% of energy metabolism is dominated by aerobic metabolism, characteristic of the low intensity and recovery phases during the match. Despite its relatively low percentage, alactic anaerobic endurance plays a very significant and crucial role, since almost all decisive actions of the match take place during efforts lasting an average of 3.5 s [2]. From the perspective of game analysis, competitive players must repeatedly hit a high-speed ball (>50 km·h^−1^) [5] more than 30 times per minute during rallies lasting no longer than 4 s, with rest periods shorter than 15 s [6]. As a result, exceptional levels of agility, reaction time, speed, strength, flexibility, and coordination are crucial to execute the techniques effectively [5,6,7,8,9].

The resistance encountered by the upper extremity when striking the ball is relatively minimal. However, the technical actions demand considerable muscular effort from the lower extremities, primarily due to the rapid acceleration and deceleration required to position and hit each ball effectively [9]. Specifically, table tennis players must perform explosive and short movements due to the frequent high-speed changes in direction during a match, which demand significant mechanical power from the extensor muscles of the lower limbs. Additionally, the high uncertainty of the opponent’s actions requires sustained and elevated muscle tension in the legs. Consequently, the explosive and reactive strength of the lower extremities is regarded as one of the key physical attributes essential for success in this sport [8,9,10].

To study the physical capacities needed by table tennis players, numerous studies have investigated the physical capacities required in table tennis players in different contexts [5,7,8]. These studies have identified differences in physical attributes such as strength and flexibility based on sex, age, anthropometric characteristics, and ranking. The findings suggest analyzing how players perform in specific tests, so coaches and physical trainers can establish benchmarks and design targeted training regimens [3]. Additionally, limb imbalances, or asymmetry, have been recognized as a potential internal risk factor for injury. As a result, analyzing asymmetries between the dominant and non-dominant limbs has become a key focus in injury prevention strategies [11]. These deficits may be associated with the demands of the sport, different training programs, or an inadequate rehabilitation process after an injury, among other factors. These asymmetries can lead to modifications in sports techniques, thereby affecting performance and increasing the risk of injury. As previously mentioned, table tennis is characterized by fast and explosive movements of the lower limbs [1,3], so an imbalance between dominant and non-dominant limbs can limit the efficiency of movements, thus, affecting the performance in this sport. Therefore, it is necessary to establish action programs to compensate for existing asymmetries and define acceptable asymmetry thresholds, making it essential to monitor unilateral lower-body strength [12].

Jump tests have long been used to measure leg power and detect imbalances between limbs in sport. While some studies have explored vertical jump performance in table tennis [3,8,9,13,14], there is limited research on gender differences in jumping ability among amateur players [8,10]. In this line, contradictory results have been found. Concretely, previous studies in table tennis have observed that male players obtained higher values in explosive movements compared to female players, analyzed with squat jump (SJ) and countermovement jump (CMJ) tests [1,3]. It seems that the differences observed between sexes may be due to better neuromuscular capacity and lower-body coordination in male players [1]. However, in another study by Pradas et al. [10], it was observed that female players obtained higher values than male players in the SJ and CMJ, with female players being able to generate higher mechanical power, but with no differences in the elasticity index [1,10]. In addition, to the authors’ knowledge, although upper-body asymmetries have been analyzed in table tennis using the handgrip test and lower body asymmetries using displacement capacity tests [1,15], with higher values observed in the dominant limb and in male players, no studies have examined lower-body asymmetries in table tennis athletes in jump tests, although this has been studied in other racket sports like tennis [16]. Closing these research gaps could have important practical benefits. For example, understanding gender-specific differences could help create customized training programs to improve performance and reduce injuries. Similarly, assessing asymmetries in young players could help identify and correct movement imbalances early, preventing long-term issues. Expanding research on metrics like the elastic index could also lead to new training methods, such as plyometric or neuromuscular exercises, to boost agility and stroke efficiency on the court. Establishing standard thresholds for asymmetries could further help coaches design personalized training plans that support long-term development. Ultimately, filling these research gaps would not only improve injury prevention and performance but also deepen our understanding of the physical demands of table tennis, helping to shape the future of player development.

Therefore, this study aimed to analyze the jump capacity and elastic index of table tennis players aged 16 to 18 based on sex. Additionally, this study assessed leg asymmetries between the dominant and non-dominant limbs during jump tests.

## 2. Materials and Methods

### 2.1. Participants

A total of 40 table tennis players (20 male and 20 female, 16.93 ± 0.80 yr, 1.72 ± 7.40 m, 65.17 ± 6.86 kg, 21.52 ± 1.54 kg/m^2^) (Table 1), who were competing during the study period in any of the Spanish official categories, aged 16 to 18, participated in this study. The inclusion criteria were (i) at least two years of national-level competitive experience, and (ii) training at least twice a week. The exclusion criteria included (i) previous injuries that could affect the study outcomes, and (ii) the use of medications. Before the commencement of the study, written informed consent was obtained from both the players and the club followed by a detailed written and oral explanation of the potential risks and benefits associated with their participation. Written informed consent from parents and assent from players were obtained for those under 18 years of age. Ethical approval was granted by the Ethics Committee for Research on Humans (CEISH, Nº 2080310018-INB0059) and the study was conducted in accordance with the Declaration of Helsinki (2013).

### 2.2. Procedures

All tests were conducted in one session. Before testing, participants completed a warm-up consisting of 5 min of forehand and backhand topspin rallies. Additionally, specific exercises were performed to familiarize them with the correct execution of the tests, including explanations and detailed corrections. The tests took place on an official table tennis court within the regular training area, during the same time frame (18:00 to 20:00) in mid-season, as teams began the second half of the league.

### 2.3. Measures

Vertical Countermovement Jump: Participants performed three consecutive vertical countermovement jumps (CMJs) with both legs and with each leg, dominant (CMJD) and non-dominant (CMJND), with 45 s rest periods in between. They were required to reach a knee angle of 90° as quickly as possible and immediately jump with their hands on the hips. Knees had to be fully extended during the jump and trials where the landing was not made with fully extended legs were repeated [3]. Flight time was measured using My Jump 2 app (version 1.3.4) [17]. The best value was used for further analysis.

Squat Jump (SJ): This jump was performed without prior countermovement, starting from a knee position of 90°. With hands placed on the hips, participants performed three consecutive SJs with both legs and with each leg, dominant (SJD) and non-dominant (SJND), with 45 s rest periods in between. Knees had to be fully extended during the jump and trials in which the landing was not executed with fully extended legs were repeated [10]. Flight time was measured using My Jump 2 app [17]. This test measures explosive strength, synchronization capacity, and instantaneous recruitment [8]. The best value was used for further analysis.

Horizontal Jump Test (HJ): With hands placed on the hips, participants performed three HJs with both legs and with each leg, dominant (HJD) and non-dominant (HJND), with 45 s rest periods in between [3]. With their hands on their hips, they jumped forward, and the distance was measured from the starting line to the landing point at heel contact using a measuring tape [3]. The best value was used for further analysis.

Elastic Index (EI): The elastic index was calculated using the following formula: ((CMJ − SJ) x 100/SJ) [10].

### 2.4. Statistical Analysis

The results are expressed as means ± standard deviation (SD). All variables followed a normal distribution, as confirmed by the Shapiro–Wilk test. A t-test for independent samples was used to assess differences between males and females in the analyzed variables. The effect size (*ES*) was calculated using Cohen’s method [18]. Effect sizes lower than 0.2, between 0.2 and 0.5, between 0.5 and 0.8, and higher than 0.8 were considered trivial, small, moderate, and high, respectively. Statistical analyses were performed using JASP software (JASP for Windows, version 0.19, Amsterdam, The Netherlands). The level of statistical significance was set at *p* < 0.05.

## 3. Results

Vertical, squat, and horizontal jump performance with both legs, dominant leg, and non-dominant leg, and elastic index results of all the table tennis and separated by gender are shown in Table 2. In all jump tests (CMJ, SJ, HJ), male players show significantly superior performance compared to female players, both with both legs and with the dominant and non-dominant legs. The effect sizes (ES) are consistently large, indicating substantial differences (*p* < 0.001, ES = 1.58 to 2.35, high). Respecting EI, contrary to the jump performance results, female players have significantly higher EI values than male players, with a very high negative effect size (*p* < 0.001, ES = −4.19, high). On the other hand, differences in jump capacity between the dominant and non-dominant legs were also observed in all jump tests.

**Figure 1:**
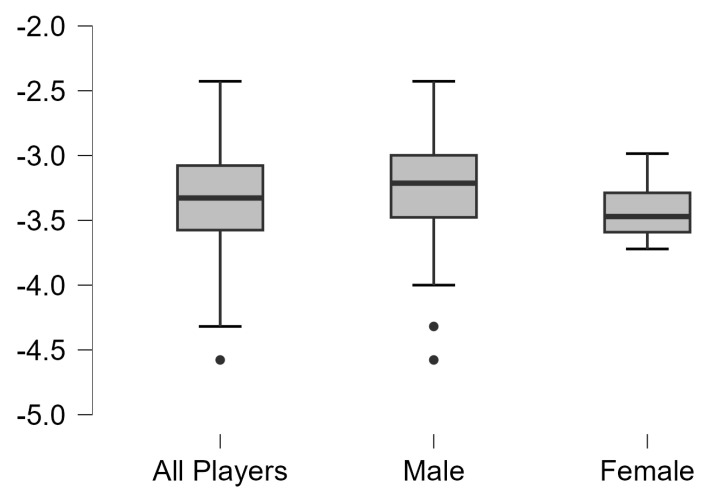
Results of asymmetries of all table tennis players and separated by gender in vertical countermovement jump test.

**Figure 2:**
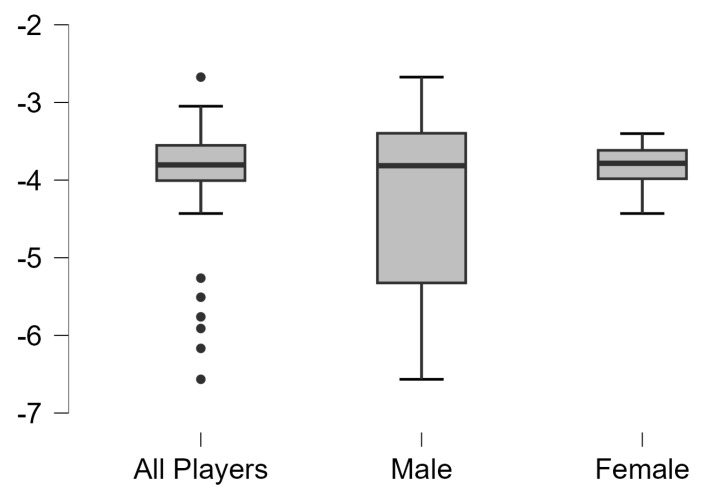
Results of asymmetries of all table tennis players and separated by gender in vertical squat jump test.

**Figure 3:**
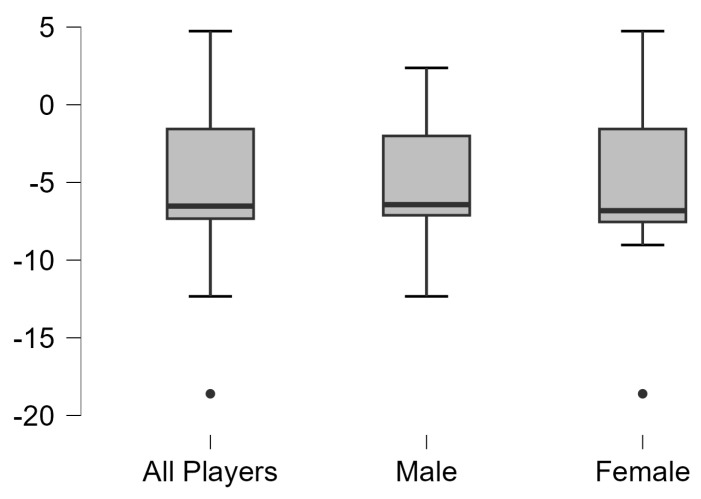
Results of asymmetries of all table tennis players and separated by gender in horizontal jump test.

Asymmetries analysis results in vertical, squat, and horizontal jump test with dominant leg and non-dominant legs of all table tennis and separated by gender are shown in Figure 1, Figure 2 and Figure 3, respectively. Significant differences were observed between dominant and non-dominant legs in vertical, squat, and horizontal jumps tests (*p* < 0.01), but no differences according to sex (*p* > 0.05).

## 4. Discussion

This study aimed to analyze the jump capacity and elastic index of table tennis players, aged 16 to 18, based on sex. Moreover, leg asymmetries between the dominant and non-dominant limbs during jump tests were also analyzed. Although previous studies have examined jump parameters in table tennis players [3,8,9,12,13], the strength of this study is the assessment of different jump tests in amateur categories. The results reveal that male players performed significantly better than female group in every test, despite having worse elastic index. On the other hand, differences in jump capacity between the dominant and non-dominant legs were also observed in all jump tests, showing significant imbalances between dominant and non-dominant legs and no differences between groups. This information could help coaches design more effective training sessions and minimize the risk of lower limb injuries by addressing leg asymmetries, ultimately optimizing performance. For instance, incorporating unilateral strength training and targeted plyometric exercises could be a valuable strategy to correct imbalances, enhance stability, and improve overall athletic performance.

Regarding the results obtained in bilateral jumps (CMJ, SJ, HJ) and EI, the results obtained in this study are partially consistent with previous studies. Concretely, Pradas et al. [8] observed better results in CMJ and SJ tests in male junior table tennis players (15–17 years), but female players tended to achieve better results in younger ages (7 to 15). In another study, Pradas et al. [9] observed that male players obtained better results in the CMJ, SJ and HJ tests than female players aged 9 to 13, but no significant differences were found. The jump capacity seems to improve as age advances, with better results for females in the early stages [7,8]. However, these differences may be influenced by age-related factors, as variations could be linked to higher plasma testosterone levels in males [8], along with differences in muscle mass development and anatomical changes in both sexes. Regarding EI, Pradas et al. [8] found that male players obtained higher values in the junior category, but no statistically significant differences were found in EI. On the other hand, Pradas et al. [10] observed that female players obtained better EI than male players, but also better jump values. However, the results of this study observed better jump capacity in male players, but lower EI. Previous studies suggest that, although female players may have a better ability to store and reuse elastic energy, this is not enough to compensate for differences in muscle strength and body mass, which favor male players in jump performance from a certain age [19]. Transferring these findings to competition, the differences in jump capacity and elastic index between male and female players could have direct implications on their playing styles and performance. According to previous studies [1,9], the fact that male players have higher jump capacity values may provide them with an advantage in the different movements performed to prepare for strokes, as well as in recovery for the next shot, directly impacting competition performance. Additionally, it has been observed that male players, due to their higher strength levels, tend to adopt a more offensive and aggressive playing style than female players, with more intense effort periods but also longer pauses between rallies [9]. On the other hand, the results obtained in this study show that female players have a higher elastic index, indicating a greater ability to reuse stored energy in the stretch-shortening cycle [8]. This energy efficiency could contribute to lower fatigue in prolonged rallies and might be related to the playing style of female players, who have shown greater game continuity and density compared to men [2].

Regarding the results obtained with one leg (CMJD, CMJDN, SJD, SJND, HJD, HJND) and leg asymmetries, to the author’s knowledge, there are no previous studies that have analyzed jumping ability unilaterally in racquet sports based on gender. However, previous studies compared jump capacity, differentiating between the dominant and non-dominant leg. Concretely, Villaplana and Blasco [19] observed that tennis players showed muscular asymmetries in their lower limbs, coinciding with the results obtained in this study. However, results that contradict those mentioned so far have been observed in other sports, such as football. Specifically, Yanci et al. [20] found significant differences, where the dominant leg showed lower strength compared to the non-dominant leg in the CMJ and HJ. On the other hand, there are also studies in which no significant differences were found in amateur football categories [21] or in healthy individuals [22] between the dominant and non-dominant leg in vertical and jumping. As suggested by previous studies [23,24], it is necessary to investigate whether the asymmetries observed between the dominant (D) and non-dominant (ND) legs are a risk factor for injury occurrence. As indicated by previous studies [21,22], there is no specific threshold in the literature to determine the point at which the risk of injury increases. However, a difference of 15% between the dominant and non-dominant leg appears to be associated with injured players or those at risk of injury, while a 10% asymmetry is linked to non-injured players or those with a low risk. Nevertheless, both the 15% threshold and lower asymmetry values should be accompanied by intervention protocols aimed at mitigating the impact of triggering factors, such as the sport itself or various training programs [21,22]. This could highlight the need for specific exercises and training protocols to reduce functional differences between both legs while enhancing explosive and elastic strength.

This study has several limitations that should be taken into account. Specifically, the primary limitation of this study is the lack of prior research that would allow a direct comparison of the results obtained. As a result, the findings had to be contrasted with studies conducted on high-level table tennis players, which could influence the outcomes since the participants in this study were from an amateur category. Furthermore, no studies were found that analyzed differences in jumping ability between the dominant and non-dominant legs in table tennis players. Therefore, the results had to be compared with research from other sports, such as tennis and football. Moreover, comparisons of our results with other studies should be cautiously approached, as the sample size and the athletes’ age could influence the results. For these reasons, future studies should focus on analyzing asymmetries between the dominant and non-dominant leg in table tennis players, evaluating their impact on performance and injury risk. In addition, it would be interesting to expand the study sample to include younger and adult categories, as well as longitudinal studies, to examine how jumping differences and asymmetries evolve throughout an athlete’s career. In addition, other variables that could influence jump capacity, such as muscle development, hormone levels, height, weight or biomechanical factors, should be taken into account. Moreover, it would be appropriate to obtain the results using a force platform. Finally, it would be valuable to design and evaluate different training programs aimed at improving jumping ability, elastic index and asymmetry levels, while assessing their effect on competitive performance.

## 5. Conclusions

The obtained results show that male table tennis players, aged 16 to 18, have better vertical and horizontal jump capacity compared to female players, despite having a lower elastic index. In addition, both male and female players show better jump capacity with the dominant leg, showing that table tennis players have asymmetries in their lower limbs. This study provides novel information by identifying significant differences in jumping ability and the elasticity index between male and female youth table tennis players. The observed differences could assist coaches in developing tailored training programs based on the sex of the players, optimizing training loads and addressing asymmetries. Specifically, detecting asymmetries between the dominant and non-dominant leg could help coaches develop specific programs to correct these imbalances, reducing injury risk and enhancing overall balance. Implementing unilateral strength training and specific plyometric exercises could be a useful strategy based on these findings. Additionally, regular jump tests could be incorporated to monitor asymmetries and apply corrective measures in a timely manner, while rehabilitation and injury prevention strategies should be developed based on the detected imbalances. To further optimize performance in competition, training programs should include exercises aimed at improving elastic energy utilization in female players to compensate for lower muscle mass. Moreover, establishing reference standards for jump capacity and elasticity index based on age and skill level would provide valuable benchmarks, allowing coaches to track progress and make data-driven decisions.

## Figures and Tables

**Table 1:** Table tennis players’ general characteristics depending on the sex (mean ± SD).

	Male	Female
Age (years)	16.90 ± 0.79	16.95 ± 0.83
Body Mass (kg)	71.20 ± 3.21	59.15 ± 3.13
Body Height (m)	1.78 ± 4.46	1.67 ± 4.90
Body Mass Index (kg/m^2^)	21.38 ± 1.83	21.65 ± 1.21

SD = Standard deviation.

**Table 2:** Results of all table tennis and separated by gender in vertical, squat, and horizontal jump test with both legs, dominant leg, and non-dominant leg with the elastic index.

	All Players	Confidence Intervals	Male	Confidence Intervals	Female	Confidence Intervals	*p* Value	ES
CMJ (cm)	39.92 ± 3.33	38.86, 40.99	42.12 ± 2.72	40.85, 43.39	37.73 ± 2.29	36.65, 38.80	<0.001	1.75
CMJD (cm)	22.41 ± 2.96	21.46, 23.35	24.30 ± 2.57	23.10, 25.51	20.51 ± 1.95	19.60, 21.42	<0.001	1.66
CMJND (cm)	21.65 ± 2.81	20.75, 22.55	23.50 ± 2.36	22.39, 24.60	19.81 ± 1.85	18.94, 20.68	<0.001	1.74
SJ (cm)	33.85 ± 3.40	32.76, 34.93	36.38 ± 2.44	35.24, 37.53	31.31 ± 2.04	30.35, 32.27	<0.001	2.25
SJD (cm)	18.09 ± 2.36	17.33, 18.84	19.55 ± 2.16	18.54, 20.57	16.62 ± 1.49	15.92, 17.32	<0.001	1.58
SJND (cm)	17.35 ± 2.16	16.66, 18.04	18.71 ± 1.90	17.83, 19.60	15.98 ± 1.43	15.31, 16.66	<0.001	1.62
HJ (cm)	202.52 ± 11.61	198.81, 206.23	210.60 ± 9.29	206.26, 214.95	194.43 ± 7.25	191.04, 197.82	<0.001	1.94
HJD (cm)	178.71 ± 14.13	174.19, 183.22	189.45 ± 9.77	184.88, 194.02	167.96 ± 8.46	164.00, 171.92	<0.001	2.35
HJND (cm)	169.53 ± 15.07	164.71, 174.34	179.58 ± 11.87	174.02, 185.14	159.47 ± 10.60	154.51, 164.43	<0.001	1.79
EI (%)	18.16 ± 2.64	17.31, 19.00	15.79 ± 1.32	15.17, 16.41	20.52 ± 0.90	20.10, 20.94	<0.001	−4.19

CMJ = vertical countermovement jump with both legs, CMJD = vertical countermovement jump with dominant leg, CMJND = vertical countermovement jump with non-dominant leg, SJ = vertical squat jump with both legs, SJD = vertical squat jump with dominant leg, SJND = vertical squat jump with non-dominant leg, HJ = horizontal countermovement jump with both legs, HJD = horizontal countermovement jump with dominant leg, HJND = horizontal countermovement jump with non-dominant leg, EI = elastic index, ES = effect size, SD = standard deviation.

## Data Availability

Due to ethical restrictions, the data supporting the findings of this study cannot be shared.
